# miRNA–221 and miRNA–483–3p Dysregulation in Esophageal Adenocarcinoma

**DOI:** 10.3390/cancers16030591

**Published:** 2024-01-30

**Authors:** Isotta Bozzarelli, Arianna Orsini, Federica Isidori, Luca Mastracci, Deborah Malvi, Marialuisa Lugaresi, Silvia Fittipaldi, Livia Gozzellino, Annalisa Astolfi, Jari Räsänen, Antonia D’Errico, Riccardo Rosati, Roberto Fiocca, Marco Seri, Kausilia K. Krishnadath, Elena Bonora, Sandro Mattioli

**Affiliations:** 1 Gastrointestinal Genetics Lab, CIC bioGUNE—BRTA, 48160 Derio, Spain; ibozzarelli@cicbiogune.es; 2 Department of Medical and Surgical Sciences (DIMEC), University of Bologna, via Massarenti 9, 40138 Bologna, Italylivia.gozzellino2@unibo.it (L.G.); marco.seri@unibo.it (M.S.); 3 Dipartimento di Genetica Medica, IRCCS Azienda Ospedaliero–Universitaria di Bologna, University of Bologna, via Massarenti 9, 40138 Bologna, Italy; federica.isidori2@unibo.it (F.I.); deborah.malvi@aosp.bo.it (D.M.); marialuisa.lugaresi2@unibo.it (M.L.); sv.fittipaldi@gmail.com (S.F.); antonietta.derrico@unibo.it (A.D.); 4 Pathology Unit, Department of Surgical Sciences and Integrated Diagnostics (DISC), University of Genoa, 16100 Genoa, Italy; mastracc@hotmail.com (L.M.); fiocca@unige.it (R.F.); 5 IRCCS Ospedale Policlinico San Martino, 16100 Genoa, Italy; 6 Institute of Oncology and Transplant Pathology, University of Bologna, 40126 Bologna, Italy; 7 Department of Cardiothoracic Surgery, University of Helsinki and Helsinki University Hospital, 00100 Helsinki, Finland; jari.rasanen@hus.fi; 8 Department of Gastrointestinal Surgery, San Raffaele Hospital, Vita–Salute San Raffaele University, 20132 Milan, Italy; rosati.riccardo@hsr.it; 9 Laboratory of Experimental Medicine and Pediatrics (LEMP), Department of Gastroenterology and Hepatology, University Hospital Antwerp, University of Antwerp, 2180 Antwerp, Belgium; sheila.krishnadath@uza.be; 10 Division of Thoracic Surgery, Maria Cecilia Hospital, 48010 Cotignola, Italy; sandro.mattioli@unibo.it

**Keywords:** esophageal adenocarcinoma, microRNA, miR–221, miR–483–3p

## Abstract

**Simple Summary:**

Low survival rates and a growing incidence characterize esophageal adenocarcinoma (EAC). MicroRNAs (miRNAs) have been linked to the development and progression of cancer, according to earlier research. Our study showed a significant correlation between poor cancer–related survival, tumor recurrence, and advanced disease stages with the overexpression of miR–221 and miR–483–3p. In particular, we have found that in low–risk EAC patients, miR–221 overexpression was linked to a lower survival rate. Therefore, these findings may help define patient stratification and determine targeted treatment for EAC.

**Abstract:**

Alterations in microRNA (miRNA) expression have been reported in different cancers. We assessed the expression of 754 oncology–related miRNAs in esophageal adenocarcinoma (EAC) samples and evaluated their correlations with clinical parameters. We found that miR–221 and 483–3p were consistently upregulated in EAC patients vs. controls (Wilcoxon signed–rank test: miR–221 *p* < 0.0001; miR–483–3p *p* < 0.0001). Kaplan–Meier analysis showed worse cancer–related survival among all EAC patients expressing high miR–221 or miR–483–3p levels (log–rank *p* = 0.0025 and *p* = 0.0235, respectively). Higher miR–221 or miR–483–3p levels also correlated with advanced tumor stages (Mann–Whitney *p* = 0.0195 and *p* = 0.0085, respectively), and overexpression of miR–221 was associated with worse survival in low–risk EAC patients. Moreover, a significantly worse outcome was associated with the combined overexpression of miR–221 and miR–483–3p (log–rank *p* = 0.0410). To identify target genes affected by miRNA overexpression, we transfected the corresponding mimic RNA (miRVANA) for either miR–221 or miR–483–3p in a well–characterized esophageal adenocarcinoma cell line (OE19) and performed RNA–seq analysis. In the miRNA–overexpressing cells, we discovered a convergent dysregulation of genes linked to apoptosis, ATP synthesis, angiogenesis, and cancer progression, including a long non–coding RNA associated with oncogenesis, i.e., MALAT1. In conclusion, dysregulated miRNA expression, especially overexpression of miR–221 and 483–3p, was found in EAC samples. These alterations were connected with a lower cancer–specific patient survival, suggesting that these miRNAs could be useful for patient stratification and prognosis.

## 1. Introduction

Esophageal adenocarcinoma (EAC) is a severe malignancy with a low survival rate and increasing incidence in Western countries. The causes of its high lethality may be attributed to inadequate screening and early diagnosis programs as well as the relative inefficiency of treatments. Indeed, most patients are diagnosed at an advanced stage, and the overall 5–year survival rate is 10–15% [1]. EAC may rise according to the widely accepted sequence of gastro–esophageal reflux disease (GERD)/intestinal metaplasia/dysplasia/adenocarcinoma, but other oncogenic pathways cannot be ruled out [2]. Since the end of the past century, several screening and early diagnosis programs promoted by scientific and professional medical societies have been implemented, particularly for patients with histologically proven Barrett’s esophagus (BE) who are thought to have a 40–50–fold higher annual incidence of EAC than the general population. However, only 12% of EAC patients have a prior diagnosis of BE, implying either difficulty in detecting BE in the diagnostic phase/surgical specimen or BE–independent pathways of EAC development [3]. The American Joint Committee on Cancer TNM staging system and the international guidelines on therapy consider EAC as a single entity [4]. However, EAC is consistently heterogeneous; therefore, different biological behaviors may impair the efficacy of unmodulated therapy [5,6,7,8]. EAC is among the tumors with the highest incidence of copy number alterations (CNAs) and somatic structural rearrangements [9]; it exhibits a high mutation frequency, and recent omics studies suggest the existence of distinct EAC subtypes based on different mutational signatures [10,11] and epigenetic mechanisms [9,10,11,12,13]. These different subtypes show a correlation with prognostic factors and potential response to therapy [13,14].

In this framework, we investigated the value of microRNA (miRNA) expression as a potential biomarker in EAC subclassification and correlation with survival. MicroRNAs (miRNAs) regulate many cell processes by binding to the 3′ untranslated region of target mRNAs and therefore modulating their expression through translational repression, mRNA degradation, or cleavage [15]. MiRNA dysregulation is implicated in different stages of tumor progression [16,17], and miRNA expression can be modulated for therapeutic purposes [18]. A few studies identified altered miRNA profiles in esophageal squamous cell carcinoma [19,20,21] and in BE–derived cancer [22,23]. However, limited knowledge exists regarding miRNAs that could discriminate among different subtypes of EAC (i.e., according to different histological subgroups).

This study aimed to assess the expression of a large number of oncology–related miRNAs in EAC samples derived from patients who underwent surgery without preoperative chemo– and radiotherapy and to correlate miRNA dysregulation with clinical features and histological subtypes to improve the efficiency of diagnosis and therapy for this aggressive form of cancer.

## 2. Materials and Methods

### 2.1. Tumor Samples

Samples for which RNA was available from formalin–fixed paraffin–embedded (FFPE) surgical resections from EAC patients among the Esophageal Adenocarcinoma Study Group Europe (EACSGE) consortium were included (124 cases) in the study. Clinical and pathological data [8,24] and EACSGE morphological classification have been previously reported [25]. The EACGSE classification was based on morphological features of esophageal/esophagogastric junction adenocarcinoma, which divided the cases into two main categories with a different prognosis: lower risk, including glandular well differentiated, mucinous muco–nodular carcinoma, and diffuse desmoplastic subgroups, and higher risk, including glandular poorly differentiated, diffuse anaplastic, invasive mucinous carcinomas, and mixed subgroups. FFPE–derived gastric mucosal samples (*n* = 8) derived from healthy individuals with no history of cancer were used as controls because we had no access to FFPE–derived samples of esophageal mucosal samples from healthy individuals. The study was approved (# L3P1223) by the Ethical Committee “Comitato Etico IRST IRCCS AVR (CEIIAV)”–Italy (Reg. Sper. 109/2016 Protocol 7353/51/2016).

### 2.2. Cell Lines

The OE19 (RRID:CVCL_1622/ECACC: 96071721) [26], OE33 (RRID:CVCL_0471), and FLO–1 (RRID:CVCL_2045) cell lines were used for functional studies. OE–19 and OE–33 cells were cultured in Roswell Park Memorial Institute (RPMI)–1640 medium (EuroClone, Milan, Italy). FLO–1 cells were cultured in high–glucose DMEM (Dulbecco’s modified Eagle’s medium). All cells were supplemented with 10% fetal bovine serum, 100 U/mL penicillin, and 100 μg/mL streptomycin (supplements from Sigma Aldrich, St. Louis, MI, USA) at 37 °C in a 5% CO_2_ atmosphere. The experiments were performed within 8 passages of resuscitation, and all experiments were performed with mycoplasma–free cells.

### 2.3. RNA Isolation from FFPE Surgical Specimens

Total RNA was isolated starting from two 10 μm thick FFPE sections enriched in the tumor area using the RecoverAll^TM^ Total Nucleic Acid Isolation for FFPE Kit (Thermo Fisher Scientific, Waltham, MA, USA) and treated with *DNase I* under RNase–free conditions, according to the manufacturer’s protocol. The yield was assessed through a NanoDrop spectrophotometer reading (Thermo Fisher Scientific), and an aliquot was run on a 1% agarose gel in 1XTBE and visualized using Midori green staining (Nippon Genetics Europe, Düren, Germany) under UV light.

### 2.4. MicroRNA Expression Profiling

The expression of 754 different human miRNAs was profiled in 8 FFPE EAC samples and a pool of 8 FFPE healthy gastric mucosal samples using the TaqMan MicroRNA Array card A2.1/B3.0 (Cat. Num. 4399966–4444303; Thermo Fisher Scientific). U6 snRNA, RNU44, and RNU48 were used as endogenous controls. Fifty nanograms of total RNA was reverse transcribed (RT) using the TaqMan microRNA Reverse Transcription Kit (Cat. Num. 00331121; Thermo Fisher Scientific) and Megaplex RT primer pools A or B (Thermo Fisher Scientific). A preamplification step was performed combining 2.5 μL of the RT reaction with the matching Megaplex PreAmp Primer Pool and TaqMan PreAmp Master Mix (Cat. Num. 4384266; Thermo Fisher Scientific) under the following conditions: 10 min at 95 °C; 2 min at 55 °C; 2 min at 72 °C; 15 s at 95 °C and 4 min at 60 °C for 12 cycles; and 99 °C for 10 min. A dilution of 1:4 in TE 0.1X was made, and 9 μL of each dilution was combined with the TaqMan Universal Master Mix, NoAmpErase UNG (2X) (Cat. Num.4440040; Thermo Fisher Scientific) and loaded on the matching TaqMan MicroRNA Array Card. The cards were run on a 7900 HT Real Time PCR system (Thermo Fisher Scientific) with the following cycling conditions: 10 min at 95 °C and 15 s at 95 °C and 1 min at 60 °C for 40 cycles. We employed the comparative 2^−ΔΔCT^ method to analyze raw data with Expression Suite software v1.0 (Thermo Fisher Scientific).

### 2.5. Single microRNA Expression Assays

The validation of miR–221 and miR–483–3p expression was performed through real–time quantitative PCR (RT–qPCR) using single TaqMan probes (Thermo Fisher Scientific). Reverse transcription was performed starting from 150 ng of total RNA extracted from FFPE sections or OE19, OE33, and FLO–1 cells using the TaqMan MicroRNA Assay (Cat. Num. 4427975; Thermo Fisher Scientific) with primers for RNU44 (#001094), miR–221 (#000524), and miR–483–3p (#002339) and using the TaqMan MicroRNA Reverse Transcription Kit with a preamplification step as described above. RT–qPCR was performed using 2 μL of the diluted preamplification product as outlined by the manufacturer. RNU44 was used as an endogenous control. For each case, the reaction was performed in triplicate. The relative miRNA expression levels for FFPE samples were calculated using the 2^−ΔΔCT^ method, comparing FFPE tumor samples versus the healthy gastric mucosal samples. For the data obtained in the single RT–qPCR assays, we applied the analysis methodology of ΔΔCT for all statistical tests. The normality and homoscedasticity of the ΔΔCT data population were verified, respectively, with the D’Agostino–Pearson normality test (the Omnibus K2 test) and the Bartlett’s test. To check the significance of the expression of the ΔΔCT of the 2 individual miRNAs compared to the pool of controls, the one—sample Wilcoxon signed–rank test was applied. The heat map was created using the Applied Biosystems™ Analysis Software using the assay–centric option. The heat map represents differences in ΔCT values compared to the ΔCT neutral/middle expression level. Thus, for each target, the middle expression level was set as the mean of all of the ΔCT values from all samples for that assay. Data can only be compared across a particular row/assay, and the colors of the boxes represent changes in ΔCT gene expression and not absolute values. Please find below a clarification on the Expression Suite Software v.1.0 (Thermo Fisher Scientific) procedure for cluster analysis and process calculation (reference DataAssist™ v2.0 Software User Instructions, https://assets.thermofisher.com/TFS–Assets/LSG/manuals/cms_094612.pdf, accessed on 20 January 2024). The heat map graphically displays results of hierarchical clustering (clustering of genes that show similar expression patterns across samples). Distances between samples and assays were calculated for hierarchical clustering based on the ΔCT values using Euclidean distance. The global control mean is the mean ΔCT value of all selected endogenous controls in the study. The colors and intensity of the boxes are used to represent changes (not absolute values) in gene expression. The scale bar is based on the ΔCT value of the neutral/middle expression level. The ΔCT value of the neutral/middle expression level is set differently such that red indicates upregulated with a ΔCT value below the middle level (thus a negative value compared to the middle expression level), and green indicates downregulated with a ΔCT value above the middle level (thus a positive value compared to the middle expression level).

miRNA expression from cell line RNA was evaluated using a commercial RNA from the normal human esophagus (Cat. Num B209050; BioChain, Newark, CA, USA).

### 2.6. miR–221 and miR–483–3p Mimic Transfection

A total of 3 × 10^5^ cells were seeded in a 6–well plate to be 80% confluent at transfection. Then, 25 nM of either mirVana™ miR–221 or miR–483–3p mimic (Cat. Num. 4464066; Thermo Fisher Scientific) and the corresponding negative controls were transfected using the TransIT–siQUEST transfection reagent (Cat. Num. MIR2114; Mirus Bio LCC; Madison USA) according to the manufacturer’s protocol. Cells were incubated at 37 °C for 48 h before RNA extraction. The validation of miR–221 and miR–483–3p overexpression was performed through RT–qPCR using single TaqMan probes, as indicated above.

### 2.7. Bulk RNA Sequencing (RNA–seq) in Transfected OE–19 Cells

RNA (250 ng) was extracted from transfected cells with a Recoverall kit (Thermo Fisher Scientific) and quantified using a NanoDrop 2000 spectrophotometer (Thermo Fisher Scientific). Library preparation and indexing for mRNA sequencing were performed with the Illumina TruSeq Stranded mRNA sample preparation kit (Illumina). Library sizes were verified using the Agilent High Sensitivity assay (Agilent Technologies, Santa Clara, CA, USA) and quantified with the dsDNA High Sensitivity Assay for Qubit v.3.0 (Thermo Fisher). All samples were equally normalized, pooled, and run on the Illumina NexSeq550, with the Mid Output Kit v2.5 flow cell (150 cycles, paired–ends). Quality control of all the generated FASTQ files was performed with FastQC [27], and the results across all samples were summarized using MultiQC [28]. Reads were mapped on the reference human genome hg38 adopting STAR [29]; duplicate removal and sorting were carried out using SAMtools [30]. Gene expression was quantified and normalized as counts per million (CPM), starting from raw gene counts generated by the python package HTseq–count [31]. In both one-to-one comparisons (OE19–C1 vs. miR–221 and OE19–C1 vs. miR–483–3p), only genes with cpm > 0 in at least one sample were selected for further analysis. The log2 ratio of each gene was calculated as the difference between the log2cpm of samples derived from the control cells transfected with a scramble sequence and the log2cpm of samples from cells transfected with the corresponding miRVana from the two miRNAs. Subsequently, miR–221 and miR–483–3p duplicates were included to perform differential gene expression (DGE) analysis using the R–bioconductor limma package [32]. Differentially expressed genes with *p* ≤ 10^−3^ were selected for the evaluation of the functional classification of biological processes and pathway overrepresentation with the analytical tools PANTHER13.1 (Protein ANalysis THrough Evolutionary Relationships; http://pantherdb.org, accessed on 20 January 2024). PANTHER is strongly connected with a variety of other genomic resources, including the UniProt Reference Proteome datasets, the Quest for Orthologues Consortium, and the InterPro Consortium of protein classification resources. In addition to the phylogenetically derived annotations, the PANTHER Overrepresentation tool also provided functional annotations directly acquired from the Gene Ontology (GO) Consortium [33].

For gene validation from RNA–seq datasets, total RNA was isolated from transfected and control cells with TRIzol (Thermo Fisher Scientific) according to the manufacturer’s instructions. Five hundred nanograms of total RNA was retrotranscribed with SuperScript IV VILO MasterMix with ezDNase Enzyme (Thermo Fisher Scientific). Ten nanograms of cDNA was used as a template for the RT–qPCRs with PowerTrack SYBR Green Master Mix 2X (Thermo Fisher Scientific) and 500 nM of both the forward– and reverse–specific primers, according to the protocol. The primers were as follows: *MALAT1* forward 5′–CGTAATGGAAAGTAAAGCCCT–3′ and reverse TCTTGTGTTCTCTTGAGGGACA; *ACTB* forward 5′–CCTGGCACCCAGCACAAT–3′ and reverse 5′–GGGCCGGACTCGTCATACT–3′. *ACTB*, encoding β–actin, was used as an endogenous control. Each reaction was performed in triplicate. Data were analyzed with the 2^−ΔΔCT^ method using total RNA from nontransfected cells as a normal control.

### 2.8. Data Analysis

Quantitative analysis of the expression data derived from the microRNA Array Card experiments was performed with Expression Suite Software v.1.0 (Thermo Fisher Scientific). The receiver operating characteristic (ROC) with the Youden index method was used to optimize the cut–off values for miRNA classification into “high–expression” and “low–expression” groups. Correlations between miRNA expression, tumor recurrence, cancer–related survival, tumor stage, and EACGSE classification were investigated using the Mann–Whitney and Kruskal–Wallis tests. Survival analysis was performed using the Kaplan–Meier method and the log–rank test. The multivariate analysis was performed according to the Cox regression analysis. The method of decision trees based on machine learning was adopted to develop a predictive algorithm of cancer–specific survival. Data analysis was performed using MedCalc 13.0.6.0 (MedCalc Software bvba, Østend, Belgium), the SPSS15.0 software package (SPSS Inc., Chicago, IL, USA), Prism (GraphPad, San Diego, CA, USA), and the R software package v.4.3.2 (R Project for Statistical Computing, Vienna, Austria). The RT–qPCR experiments were analyzed with Student’s *t*–test and one–way ANOVA. *p* values < 0.05 were considered statistically significant.

The data supporting this study findings are available from the corresponding author upon request.

## 3. Results

### 3.1. Discovery Dataset: Identification of Deregulated miRNAs in EAC

**Figure 1:**
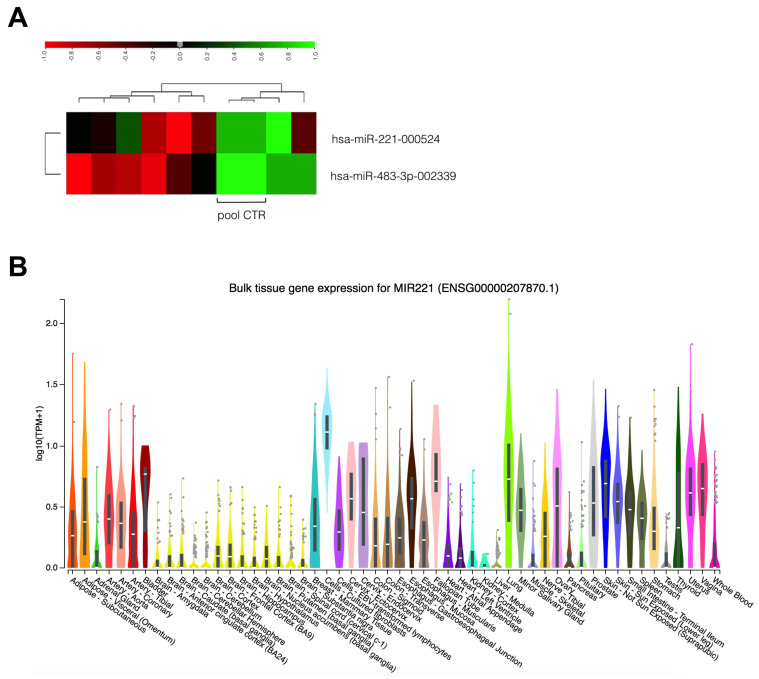
Discovery of deregulated miRNAs in EAC. (**A**) Heatmap target centric, showing the differential expression of hsa (*Homo sapiens*) miR−221 and miR−483−3p in EAC samples and control samples (pool CTR). For each target, the middle expression level is set as the mean of all of the ΔCT values from all samples for that assay. Data can only be compared across a particular row. The color and intensity of the boxes are used to represent changes (not absolute values). Red indicates upregulated with a ΔCT value below the middle level (thus a negative value compared to the middle expression level), and green indicates downregulated with a ΔCT value above the middle level (thus a positive value compared to the middle expression level). Scale bar represents ΔCT values. (**B**) Expression of miR–221 in different human tissues, image derived from the Genotype−Tissue Expression (GTEx) portal.

To study the differential expression of 754 tumor–related miRNAs, we first profiled eight EAC samples vs. eight healthy tissue samples. Gene expression analysis revealed several dysregulated miRNAs with a significant adjusted *p* < 0.05 (Supplementary Table S1). Among them, miR–221 and miR–483–3p were consistently overexpressed, with mean fold increases of 2.746 and 11.33, respectively (Figure 1A). We verified the expression of miR–221 and miR–483–3p in human tissues in the Genotype–Tissue Expression (GTEx) portal: miR–221 was expressed in different tissues, including the esophagus, where it was more highly expressed in the esophageal mucosa compared to the esophageal junction (Figure 1B). A very low expression of miR–483–3p was identified in normal tissues (Supplementary Figure S1).

### 3.2. Replication Dataset (EACGSE Cohort): Single miRNA Analysis

**Figure 2:**
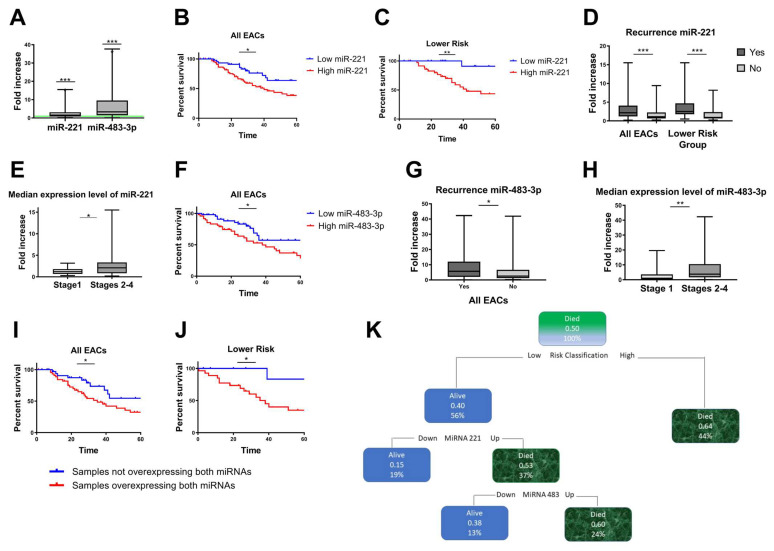
miR–221 and miR–483–3p were significantly upregulated in the EAC replication group. (**A**) miR–221 and miR–483–3p expression levels in a cohort of 124 EAC patients (All EACs). The values are expressed as the fold increase compared to control FFPE–derived healthy gastric tissue samples (green baseline) (Wilcoxon signed–rank test, *p* < 0.0001). (**B**,**C**) miR–221 expression levels and correlation with clinical outcomes**.** Kaplan–Meier curves show the cancer–specific survival for EAC groups stratified by miR–221 expression levels. (**B**) All EAC cases (log–rank *p* = 0.0025). (**C**) EACSGE lower–risk subgroup (log–rank *p* = 0.0065). Blue line: samples with low expression of miR–221; red line: samples with high expression of miR–221. (**D**) Correlation between miR–221 expression and recurrence in All EACs (Mann–Whitney *p* = 0.0002) in the lower–risk EACSGE subgroup (Mann–Whitney *p* = 0.0005). (**E**) Correlation between miR–221 expression and TNM stages (Mann–Whitney *p* = 0.0195 stage 1 versus stage 2–3–4). (**F**) miR–483–3p expression levels and correlation with clinical outcomes. Kaplan–Meier curves show cancer–specific survival for EAC groups stratified based on miR–483–3p expression levels in All EAC patients (log–rank *p* = 0.0235). (**G**) Recurrence in All EACs (Mann–Whitney *p* = 0.0173). (**H**) Correlation between miR–483–3p and TNM stages (Mann–Whitney *p* = 0.0085 stage 1 versus stage 2–3–4). (**I**,**J**) Combined overexpression of miR–221 and miR–483–3p and correlation with survival. Kaplan–Meier curves for patients overexpressing both miRNAs versus patients not overexpressing both miRNAs showing cancer–specific survival in (**I**) All EAC cases (log–rank *p =* 0.0410) and (**J**) lower–risk EACSGE (log–rank *p* = 0.0340). (**K**) Predictive algorithm of cancer–specific survival. By using the decision tree method, a predictive algorithm of cancer–specific survival was developed. * = *p* ≤ 0.05; ** = *p* ≤ 0.01; *** = *p* ≤ 0.001.

We investigated the expression of miR–221 and miR–483–3p in a separate cohort of 124 RNAs derived from FFPE surgical specimens from EAC patients (Supplementary Table S2). In accordance with our preliminary array data, these two miRNAs were significantly overexpressed in EAC tissues compared to normal tissues (miR–221 mean fold increase 2.276, Wilcoxon signed–rank test: *p* < 0.0001; miR–483–3p mean fold increase 5.964 *p* < 0.0001; Figure 2A).

### 3.3. Correlation between miRNA–221 Expression and EAC Clinicopathological Features

We studied the relationship between miR–221 dysregulation and several clinical parameters in EAC samples. Using ROC curve analysis and the Youden index approach, the 124 EAC patients were categorized into “high” and “low” miR–221 expression groups (cut–off value of 1.32–fold change, *p* = 0.003, Supplementary Figure S2A). Patients with high levels of miR–221 expression had a considerably worse prognosis, according to Kaplan–Meier curves for cancer–specific survival (log–rank *p* = 0.0025, Figure 2B).

Next, we sought to correlate miRNA expression with EAC morpho–functional characteristics using the recently published EACSGE classification [25]. We found a significant correlation between miR–221 overexpression and worse outcome in the lower–risk subgroup (log–rank *p* = 0.0065; Figure 2C), whereas there was no significant correlation in the higher–risk subgroup (Supplementary Figure S3A). Moreover, when considering all EAC cases, higher median expression levels of miR–221 were observed in relapsed patients than in nonrelapsed patients, but this association was more significant in the lower–risk subgroup (Mann–Whitney test *p* = 0.0005 and *p* = 0.0002, respectively, Figure 2D). In comparison to stage I patients, those with advanced disease stages (stages II–IV) had significantly greater expression levels of miR–221 (Mann–Whitney test *p* = 0.0195, Figure 2E).

### 3.4. Correlation between miRNA–483–3p Expression and EAC Clinicopathological Features

We evaluated the correlation between miR–438–3p expression and clinical variables in our EAC cohort. All cases were divided into “high” or “low” miRNA–483–3p expression groups by evaluating the ROC curve with the Youden index method (cut–off value of 3.15–fold–change, *p* = 0.0295, Supplementary Figure S2B).

In all EAC cases, patients with high miR–483–3p expression levels had a worse prognosis, according to Kaplan–Meier analysis for cancer–specific survival (log–rank *p* = 0.0235; Figure 2F), but it was not possible to observe specific differences in EACGSE lower– vs. higher–risk groups (Supplementary Figure S3B,C, respectively).

Patients with relapses had significantly higher median expression levels of miR–483–3p (Mann–Whitney test *p* = 0.0173; Figure 2G). For patients overexpressing miR–483–3p, we also discovered a significant expression increase from stage I to later stages (Mann–Whitney *p* = 0.0085; Figure 2H).

### 3.5. Concurrent miRNA–221 and 483–3p Overexpression Is Correlated with Poor Survival

To assess the combined effect of miR–221 and miR–483–3p on cancer–related survival, Kaplan–Meier analysis was used to compare patients with both miRNAs overexpressed versus the rest of the EAC patients (i.e., either only one miRNA overexpressed or both not overexpressed). We found that the combined overexpression of miR–221 and miR–483–3p was linked to a significantly worse outcome in all EAC patients, particularly in the lower–risk EACGSE subgroup (log–rank *p* = 0.0410 and *p* = 0.0340, respectively, Figure 2I,J).

Multivariate Cox regression analysis identified as statistically significant predictive variables for cancer–specific survival the histological classification in low–risk and high–risk groups of EAC (*p* < 0.0001, HR 3.282, 95% CI 1.842–5.846) and the pathological stage (*p* = 0.031, HR 9.279, 95% CI 1.229–70.056). The analysis to recognize a predictive prognostic value for miRNA dysregulation showed a trend toward significance (overexpression of miRNA–221, *p* = 0.071).

Nonetheless, when we used a predictive algorithm of cancer–specific survival developed using decision trees, the developed algorithm selected only the histological classification of EAC and the dysregulation of miR–221 and miR–483–3p in relation to cancer–specific survival (Figure 2K).

### 3.6. miRNA Overexpression and Transcriptome Analysis In Vitro

**Figure 3:**
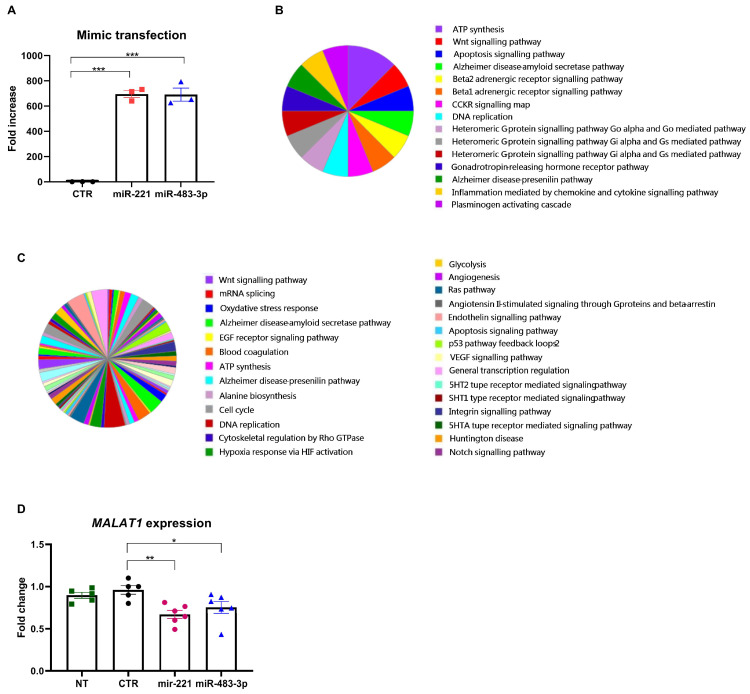
Differentially expressed genes (DEGs) in transfected OE19 cells. (**A**) OE19 cells were transfected with either miR–221 or miR–483–3p mimic and a scramble negative control (CTR). The transfection efficiency was evaluated by RT–qPCR for miR–221 and miR–483–3p (ANOVA test *p* < 0.0001 and *p* < 0.0001, respectively). After transfection, RNA–seq analysis of DEGs was performed vs. cells transfected with negative control. Biological processes of genes (GO–Slim Biological processes) differentially expressed in EAC, as identified by PANTHER Functional Classification analysis, are reported for (**B**) mir–221 and for (**C**) mir–483–3p. (**D**) Real–time qRT–PCR data for *MALAT1* expression. Data from transfected cells (overexpressing either miR–221 (pink circle) or miR–483–3p (blue triangle) were compared vs. cells transfected with a negative control CTR (black circle) and with untransfected cells (NT, green square) (normalization was performed on a commercial pool of esophageal control tissues); human beta–actin was used as an endogenous control gene (ANOVA test; *p* = 0.007 and *p* = 0.0469).* *p* < 0.05; ** *p* < 0.01; *** *p* < 0.0001.

We evaluated miR–221 and miR–483–3p expression in three different EAC cell lines. Data were normalized using the RNA derived from a commercial pool of fresh normal human esophageal tissues [34]. Basal expression was present for both miRNAs, with no significant differences in the three cell lines regarding miR–221. However, an increased expression of miR–483–3p could be observed in FLO–1 cells, although not reaching statistical significance (Supplementary Figure S4A,B). Therefore, we selected the OE–19 cell line for further experiments to investigate the targets of miR–221 and miR–483–3p since it carries a *TP53*–inactivating mutation and *ERBB2* amplification, conditions present in several of the EAC cases included in the analysis [11,24]. OE–19 cells were transfected with either miR–221 or miR–483–3p mimic and a scramble negative control. Total RNA was extracted 48 h post–transfection, and transfection efficiency was evaluated by RT–qPCR for miR–221 and miR–483–3p, normalizing the expression with the endogenous control RNU44, using the scramble–transfected cells as controls (ANOVA test *p* < 0.0001 and *p* < 0.0001, respectively (Figure 3A). Next, we assessed the overall impact of miR–221 and 483–3p overexpression on gene expression via RNA–seq. RNA–seq was carried out on two independent transfected samples for each miRNA and scramble transfection. Data filtering, annotation, and comparison were carried out according to our published pipeline [11]. Quantitative data analysis identified 220 altered genes with differential expression between miRNA–transfected and scramble–transfected cells when miR–221 was overexpressed (*p* ≤ 10^−3^, Supplementary Table S3). A total of 868 altered genes were identified when miR483–3p was overexpressed (*p* ≤ 10^−3^, Supplementary Table S4). Notably, the majority of the dysregulated genes were noncoding genes, such as long non–coding RNAs (lncRNAs), miRNAs, and small nucleolar RNAs (snoRNAs), suggesting an important role of these miRNAs in the regulation of transcriptional complexes in EAC.

Analysis using the PANTHER Functional Classification test (PANTHER GO–Slim Process) for miR–221 led us to functionally map a total of 159 out of 220 differentially expressed genes to different biological processes, including ATP synthesis, the Wnt signaling pathway, p53 pathways, apoptosis, inflammation, and neurodegenerative disorders (Figure 3B).

For miR–483–3p, we were able to map 172 of the 868 differentially expressed genes. Pathway analysis revealed that angiogenesis, Notch and Ras signaling, and cell cycle regulation were the main enriched pathways (Figure 3C). Several pathways were shared between the two miRNAs, suggesting a possible convergence in regulating oncogenic pathways.

Among the shared dysregulated genes, *MALAT1* (metastasis–associated lung adenocarcinoma transcript 1) [35] was downregulated in both miR221 and miR483–3p overexpression analysis (*p* = 3.32 × 10^−55^ and *p* = 3.52 × 10^−21^, respectively). Thus, we further investigated *MALAT1* via RT–qPCR in transfected OE–19 cells vs. those transfected with the scramble sequence. RNA from untransfected cells was used for data normalization using the ΔΔCt method. As reported in Figure 3D, we confirmed the dysregulated expression of *MALAT1* in cells overexpressing either miR221 or miR483–3p (ANOVA test, *p* = 0.007 and *p* = 0.0469, respectively).

## 4. Discussion

EAC is characterized by high aggressiveness and poor prognosis [36,37]. The possibility of applying highly powered prognostic algorithms based on pathology and biomolecular patterns would have a capital role in tailoring therapies according to biological characteristics of EAC and in driving an appropriate choice and timing of therapeutic and surgical options to improve their efficacy. EAC biological heterogeneity might be a barrier to achieving this fundamental goal. Several cancer–related characteristics are accelerated by EAC genomic instability, and some acquired mutations can confer benefits to altered cells in specific ways [38]. However, when a cancer genome is very heterogeneous, as in EAC, it is difficult to fully characterize all the mutations, chromosomal rearrangements, and epigenetic changes that give rise to tumor development and progression [38,39]. In our recent study, we showed that a combination of high–throughput sorting technology and massive sequencing could lead to a better definition of the EAC mutational status and inter– and intratumor heterogeneity than analysis of whole–tumor samples [11]. The identification of more/better prognostic markers, however, would help to subclassify the different forms of EACs since the number of drivers per sample is frequently insufficient to fully explain the disease. Our most recent research developed a diagnostic algorithm that classified specific histotypes from adenocarcinomas with glandular architecture, further grading the former and subclassifying the latter. When combined with stage, this morphological differentiation was shown to have a statistically significant prognostic influence either on its own or when dichotomized into lower– and higher–risk carcinomas. Indeed, the stage plus combination showed a high discriminating power for five–year cancer–specific survival [25].

It is a well–known concept that miRNAs can play an active role in tumor development and progression [16,40]. Specific miRNA signatures have been identified and translated into clinically relevant diagnostic and prognostic markers in thyroid cancer and hematological diseases [41,42]. In our study, we found two dysregulated miRNAs, miR–221 and miR–483–3p, that were reproducibly overexpressed in EAC samples. Their overexpression had previously been reported in many human cancers, and in vitro and in vivo studies supported a causal role for tumor progression according to their dysregulated expression. In particular, in hepatocellular carcinoma, miR–221 overexpression correlates with tumor aggressiveness in terms of the number of metastases and multifocal lesions [43]. A possible role of miR–221 in EAC progression has been provided by Matsuzaki and colleagues since they reported an increased level of miR–221/222 in EAC compared to the surrounding BE [44]. Furthermore, in EAC, miR–221 is involved in 5–fluorouracile (5–FU) chemoresistance, leading to alteration of the Wnt/β–catenin pathway [45]. In our EAC cohort, we discovered an inverse relationship between increased miR–221 expression and cancer–specific survival and a significant correlation between increased miR–221 expression and tumor recurrence. Moreover, when we evaluated miRNA expression within the framework of the EACGSE categorization that distinguishes different histotypes [25], we observed that in lower–risk carcinomas, patients with high levels of miR–221 expression had inferior cancer–specific survival and a significant correlation with recurrence. The correlation between EACGSE lower and higher risk, miR–221 overexpression, and prognosis was further corroborated by the analysis using a predictive algorithm of cancer–specific survival.

The *hsa–mir–483* gene (encoding both miR–483–5p and miR–483–3p) is a mammalian–conserved microRNA located within intron 2 of the human insulin growth factor 2 (IGF2) locus [46], an imprinted gene. Defects in the imprinting of the IGF2 locus are observed in Beckwith–Wiedemann syndrome, characterized, among other features, by an increased incidence of pediatric malignancies (nephroblastoma or Wilms’ tumor, hepatoblastoma, and rhabdomyosarcoma) [47]. miR–483–3p is overexpressed in Wilms’ tumors [48] but also in adult cancers such as colon, breast, and hepatocellular carcinoma [49,50,51]. In our EAC cohort, a lower expression of miR–483–3p was found in EAC tumors at stage I compared to other more advanced stages, suggesting that the miR–483–3p signature might also be useful for patient stratification.

To identify target genes modulated by miRNA upregulation, we enhanced the expression of miR–221 and miR–483–3p in OE–19 cells, a cell model of EAC that carries both a loss–of–function *TP53* mutation and *ERBB2* amplification. We performed a transcriptome analysis to identify the differentially expressed genes in cells overexpressing the two different miRNAs vs. OE19 cells transfected with a control negative sequence. Among the transcripts that exhibited significant dysregulation, many were noncoding genes, suggesting a complex regulatory system influenced by these miRNAs and targeting the transcription regulatory machinery. Among the protein–coding genes that we found dysregulated, several genes of interest have already been reported in the literature to be associated with cancer. For instance, in cells overexpressing miR–221, we observed an upregulation of *FRAT2*, *AMD1*, and *MTHFD1L*, genes linked to tumor progression, severity, invasiveness, and worse prognosis [52,53,54,55,56,57,58,59,60]. *ENTP6* was found to be downregulated, as previously reported in testicular cancer associated with cisplatin resistance [61].

It is interesting to identify *MALAT1*, a long non–coding RNA involved in cancer metastasis, as a target of both miR221 and miR483–3p overexpression. *MALAT1* is aberrantly expressed in pancreatic cancer, lung cancer, breast cancer, colorectal cancer, gastric cancer, nasopharyngeal carcinoma, hepatocellular carcinoma, and osteosarcoma [62]. *MALAT1* is a nuclear–enriched and highly conserved lncRNA abundantly expressed in cells and tissues and is involved in mitochondrial homeostasis, cell proliferation, and apoptosis. It has been shown that in lung epithelia, *MALAT1* downregulation leads to reduced apoptosis and promotes cell viability [63], suggesting a context–dependent regulation of different cell processes for this lncRNA. It will be interesting to further evaluate the role and expression of *MALAT1* in additional independent EAC samples [26,34].

Overall, it will be of key importance to expand the analysis of the expression of miR221 and miR483–3p in independent cohorts of EAC patients, also in the context of circulating fluid (liquid biopsy) testing. Indeed, liquid biopsy has emerged as a promising tool for diagnosis, prognosis, and patient stratification for personalized therapy for various solid tumors. Therefore, combining this molecular approach with clinical parameters could help stratify EAC patients to improve their management with tailored therapies.

## 5. Conclusions

In conclusion, the study of miR–221 and miR–483–3p expression in our EAC cohort revealed that they are significantly overexpressed, and this dysregulation is correlated with worse clinical parameters. RNA sequencing analysis has demonstrated that this dysregulation leads to differential expression of genes previously reported to have a role in cancer development and progression.

Moreover, miR–221 profiling seems to be a promising strategy to identify patients with worse survival, especially in the EACGSE lower–risk group, providing a valuable molecular parameter to stratify EAC patients.

It will be important to characterize the expression of these miRNAs among circulating fluids in EAC patients (liquid biopsy). Indeed, liquid biopsy has emerged as a promising tool for diagnosis, prognosis, and patient stratification related to personalized therapy for various solid tumors. Therefore, combining this molecular approach with clinical parameters could help stratify EAC patients to improve their management and address specific therapeutic options and targets for tailored therapies.

## Data Availability

All data are available from the authors upon request.

## Supplementary Materials

The following supporting information can be downloaded at: https://www.mdpi.com/article/10.3390/cancers16030591/s1, Figure S1: Differential expression of miR–483 in human tissues, image from the Genotype–Tissue Expression (GTEx) portal. Figure S2. ROC curve with Youden index method was used to optimize cut–off values for miRNAs classification into a “high expression” and “low expression” groups. A. miR–221 (cut–off value of 1.32 fold–change; *p* = 0.003); B. miR–483–3p (cut–off value of 3.15 fold–change; *p* = 0.0295). Figure S3. Kaplan–Meier curves show cancer–related survival for A. EACSGE Higher risk subgroup stratified based on miR–221 expression levels; B–C. EACSGE Lower (B) and Higher (C) risk subgroup stratified based on miR–483–3p expression levels. Figure S4: MiRNA expression levels in OE–19 EAC cell lines. A. Expression levels in OE–19 and FLO–1 EAC cell lines of miR–221 and miR–483–3p compared to esophageal healthy control tissue. B. mirVana 221 mimic and 483–3p mimic transient transfection efficiency was evaluated with RT–qPCR using TaqMan single assay for miR–483–3p normalized with RNU44 (ANOVA test *p* < 0.0001 and *p* < 0.0001). Table S1: List of miRNAs differentially expressed in 8 EAC cases vs. 8 healthy tissues. Table S2: Clinical and epidemiological data for the EAC cases included in the study. Sex (0 = Male 1 = Female), age, Cancer Specific Survival (CSS, 1 = Death), follow up (months), stage (1–4), EACSGE classification (L = Lower Risk; H=Higher Risk). Table S3: List of target genes potentially dysregulated by miR–221 expression according to literature. Table S4: List of target genes potentially dysregulated by miR–483–3p expression according to literature
