# Supplementary material for: miRNA–221 and miRNA–483–3p Dysregulation in Esophageal Adenocarcinoma
Source: Cancers (Basel). 2024 Jan 30;16(3):591. doi: 10.3390/cancers16030591 (PMC10854562; doi:10.3390/cancers16030591)
Supplement: Supplementary file 1 [file cancers-16-00591-s001.zip › cancers-2786770-supplementary - all.zip!/cancers-2786770-supplementary - all.pdf]

Supplementary Materials

Table S1: List of miRNAs differentially expressed in EAC. Results from the expression data analysis with the TaqMan MicroRNA Array cards in 8 EAC cases compared to two pools of 8 controls. Up-regulated miRNAs are highlighted in orange and down-regulated in grey. The corresponding fold changes and adjusted P are indicated for each dysregulated miRNA.

Table S2: Clinical and epidemiological data for the EAC cases included in the study. Sex (0=Male 1=Female), age, Cancer Specific Survival (CSS, 1=Death), follow up (months), stage (1-4), EACSGE classification (L= Lower Risk; H=Higher Risk).

Table S3: List of target genes dysregulated by miR-221 overexpression in OE-19 cell lines.

Table S4: List of target genes dysregulated by miR-483-3p overexpression in OE-19 cell lines.

Supplementary Figure Legends

Figure S1: **Expression of miR-483 in human tissues**. Image derived from the Genotype-Tissue Expression (GTEx) portal.

Figure S2. **ROC curve.** ROC curve was calculated with Youden index method was used to optimize cut-off values for miRNAs classification into a "high expression" and "low expression" groups. (A). miR-221 (cut-off value of 1.32 fold-change; P=0.003); (B). miR-483-3p (cut-off value of 3.15 fold-change; P=0.0295).

Figure S3. Kaplan-Meier cancer-specific survival and miRNA expression. Curves show cancer-specific survival for (A) EACSGE Higher risk subgroup stratified based on miR-221 expression levels; (B- C). EACSGE Lower (B) and Higher (C) risk subgroup stratified based on miR-483-3p expression levels.

Figure S4: **MiRNA expression levels in EAC cell lines**. (A). Expression levels in OE-19, OE-33 and FLO-1 EAC cell lines of miR-221 and (B) miR-483-3p compared to esophageal healthy control tissue.
